# Single Prolonged Stress Reduces Intrinsic Excitability and Excitatory Synaptic Drive Onto Pyramidal Neurons in the Infralimbic Prefrontal Cortex of Adult Male Rats

**DOI:** 10.3389/fncel.2021.705660

**Published:** 2021-07-23

**Authors:** Nawshaba Nawreen, Mark L. Baccei, James P. Herman

**Affiliations:** ^1^Department of Pharmacology and Systems Physiology, University of Cincinnati, Cincinnati, OH, United States; ^2^Neuroscience Graduate Program, University of Cincinnati, Cincinnati, OH, United States; ^3^Cincinnati Veterans Affairs Medical Center, Cincinnati, OH, United States; ^4^Department of Anesthesiology, Pain Research Center, University of Cincinnati Medical Center, Cincinnati, OH, United States

**Keywords:** prefrontal cortex, excitability, single prolonged stress, synaptic inputs, GABA, glutamate

## Abstract

Post-traumatic stress disorder (PTSD) is a chronic, debilitating mental illness marked by abnormal fear responses and deficits in extinction of fear memories. The pathophysiology of PTSD is linked to decreased activation of the ventromedial prefrontal cortex (vmPFC). This study aims to investigate underlying functional changes in synaptic drive and intrinsic excitability of pyramidal neurons in the rodent homolog of the vmPFC, the infralimbic cortex (IL), following exposure to single prolonged stress (SPS), a paradigm that mimics core symptoms of PTSD in rats. Rats were exposed to SPS and allowed 1 week of recovery, following which brain slices containing the PFC were prepared for whole-cell patch clamp recordings from layer V pyramidal neurons in the IL. Our results indicate that SPS reduces spontaneous excitatory synaptic drive to pyramidal neurons. In addition, SPS decreases the intrinsic membrane excitability of IL PFC pyramidal cells, as indicated by an increase in rheobase, decrease in input resistance, hyperpolarization of resting membrane potential, and a reduction in repetitive firing rate. Our results suggest that SPS causes a lasting reduction in PFC activity, supporting a body of evidence linking traumatic stress with prefrontal hypoactivity.

## Introduction

Post-traumatic stress disorder (PTSD) is among the most prevalent and debilitating neuropsychiatric disorders in the world. In the United States alone, nearly 25 million people will develop PTSD at some point in their lives (Kessler et al., 1995; Maren and Holmes, 2016). To date no universally efficacious treatment exists for PTSD, underscoring the importance of the development of effective therapeutic strategies for this disorder.

Clinical research has linked PTSD with deficits in fear extinction (Ressler et al., 2004; Quirk et al., 2006; Milad et al., 2008), indicative of enhanced responsiveness to emotional stimuli. Symptoms of PTSD can be modeled in rodents using the single prolonged stress (SPS) paradigm (Liberzon et al., 1997; Eagle et al., 2013). For example, multiple studies indicate that SPS disrupts the extinction of fear memories in male rats (Yamamoto et al., 2008; Knox et al., 2012; Souza et al., 2017). Studies in our group indicate that SPS disrupts extinction learning in male (but not female) rats, indicative of a sex-specific impact on fear regulation (Cotella et al., 2021).

Under normal conditions of fear extinction, excitatory projections from the infralimbic (IL) medial prefrontal cortex (mPFC) to the basolateral amygdala (BLA) activate the intercalated cell clusters, which ultimately reduces output from the central amygdala, thereby promoting extinction of fear (Garcia et al., 1999; Milad and Quirk, 2002; Quirk et al., 2003; Likhtik et al., 2005). However, exposure to an acute traumatic experience may lead to a reduction in activity in the IL mPFC, resulting in a loss of prefrontal inhibition of the amygdala, consistent with exaggerated fear responses (Akirav and Maroun, 2007).

The IL plays an essential role in extinction, extinction recall and reinstatement of conditioned fear, and damage or inactivation of this structure produces extinction memory deficits that resemble those seen in PTSD patients (Sotres-Bayon and Quirk, 2010; Norrholm et al., 2011; Sierra-Mercado et al., 2011). Indeed, abnormally low mPFC activity, together with abnormally high amygdala activity, can be observed in PTSD patients (Liberzon et al., 1999; Milad et al., 2009). Magnetic resonance spectroscopy (MRS) studies indicate that activation of the IL mPFC is reduced following SPS in rats (Piggott et al., 2019), further consistent with a role for reduced IL output in stress pathology. While the role of the IL in promoting fear extinction and emotional regulation is well documented (Sierra-Mercado et al., 2011; Milad and Quirk, 2012; Cho et al., 2013; Little and Carter, 2013; Kim et al., 2016), the cellular mechanisms underlying severe stress-related dysfunction remain to be determined.

In this study we tested the impact of SPS on electrophysiological properties of principal projection neurons in layer V of the IL, assessing (1) intrinsic membrane excitability (2) excitatory and inhibitory synaptic drive. Our results indicate that SPS reduces the intrinsic excitability of IL projection neurons and decreases the efficacy of excitatory signaling onto this population, the latter likely reflecting a reduction in presynaptic glutamate release. We also observed that SPS slowed the decay of GABAergic currents, although this was insufficient to significantly change the overall inhibitory synaptic drive onto IL-PFC neurons. Our results highlight a novel potential mechanism underlying the reduced prefrontal activity observed following SPS, and provides insight into the pathophysiology of abnormal fear memory deficits associated with PTSD.

## Methods

### Rats

Male Sprague Dawley rats were purchased from Envigo and allowed to acclimate for a week at the University of Cincinnati animal housing facility. Male rats were used for the study as prior studies in our group indicate SPS-induced extinction learning deficits in male rats only (Cotella et al., 2021). Rats were maintained under standard conditions (12/12 h light/dark cycle, 22 ± 1°C, food and water *ad libitum*; two rats per cage) in accordance with the University of Cincinnati Institutional Animal Care and Use Committee, which specifically approved all stress regimens employed in this study. All animal experiments were carried out in accordance with the National Institutes of Health Guide for the Care and Use of Laboratory Animals (NIH Publications No. 8023, revised 1978). All experiments were performed on adult male rats at 12 weeks of age.

### Single Prolonged Stress (SPS) Protocol

Animals were randomly assigned into the control or SPS groups. After the acclimation period, the SPS group was exposed to the SPS paradigm. SPS consisted of three sequential stressors (restraint stress, forced swimming, and ether exposure). First, rats were restrained for 2 h in a plastic animal restrainer, followed immediately by 20 min of forced group swim in water (20–24°C) in a tub, filled two-thirds from the bottom. Following 15 min of recuperation, rats were exposed to ether vapors (inside a desiccator) until loss of consciousness (less than 5 min). Rats were then returned to their home cages for 7 days without further disturbance (Knox et al., 2012). Control group rats remained in their home cages for 7 days without any stress.

### Electrophysiology

#### Slice Preparation

Rats were sacrificed 7 days post SPS at approximately postnatal day 91. The 7 days incubation time was selected as behavioral abnormalities are consistently observed following this incubation period (Iwamoto et al., 2007; Knox et al., 2012, 2016; Keller et al., 2015). Animals were deeply anesthetized with sodium pentobarbital (390 mg/kg, Fatal-Plus) and decapitated. A warm slicing protocol was used to prepare healthy adult rat brain slices as previously described (Ting et al., 2014). Adult rats of approximately 12 weeks of age were used for electrophysiology, since SPS effects on fear learning are observed in adult rats (Knox et al., 2012) (adolescent animals are resistant to the effects of SPS and do not show deficit in fear extinction) (Chen C. V. et al., 2018). Brains were quickly isolated and dura matter carefully removed before removing the cerebellum. The brain was then immediately glued to a cutting stage and immersed in NMDG solution (92 mM NMDG, 2.5 mM KCl, 1.2 mM NaH_2_PO_4_, 30 mM NaHCO_3_, 20 mM HEPES, 25 mM glucose, 5 mM sodium ascorbate, 2 mM thiourea, 3 mM sodium pyruvate, 10 mM MgSO_4_, and 0.5 mM CaCl_2_) at a temperature of 34–36°C and continuously bubbled with 95% oxygen and 5% carbon-dioxide. Coronal slices containing the IL mPFC were sectioned at 300 μm thickness using a vibrating microtome (Vibratome 7000smz-2; Campden Instruments Ltd., Lafayette, IN, United States) with ceramic blades (Campden Instruments Ltd.) at an advance speed of 0.03 mm/s. Vertical vibration of the blade was manually tuned in accordance with the user manual, and was set to 0.1–0.3 μm. Bath temperature was kept within the desired range of 34–36°C, by adding warm or cold water into the external chamber of the Vibratome, and was monitored throughout the cutting procedure with a conventional mercury/glass thermometer. The slices were allowed to recover for 1 h in oxygenated NMDG solution at 34–36°C. At the end of recovery, slices were transferred to a chamber containing oxygenated artificial CSF solution (125 mM NaCl, 2.5 mM KCl, 25 mM NaHCO_3_, 1 mM NaH_2_PO_4_, 25 mM glucose, 1 mM MgCl_2_, and 2 mM CaCl_2_) for at least 30 min at room temperature after which the slices were ready for *in vitro* patch clamp recordings.

#### Electrophysiological Recording From Layer V IL mPFC

Brain slices were transferred to a submersion-type recording chamber (RC-22; Warner Instruments, Hamden, CT, United States) and mounted onto the stage of an upright microscope (BX51WI, Olympus, Center Valley, PA, United States). Slices were then perfused at a flow rate of 2–4 ml/min with oxygenated aCSF at 34–36°C. Patch electrodes were constructed from thin-walled single-filamented borosilicate glass (1.5 mm outer diameter; World Precision Instruments) using a microelectrode puller (P-97; Sutter Instrument, Novato, CA, United States). Pipette resistances ranged from 4 to 6 MΩ, and seal resistances were >1 GΩ.

Whole-cell patch clamp recordings were obtained from layer V pyramidal neurons in the IL mPFC using a MultiClamp 700B amplifier (Molecular Devices, Sunnyvale, CA, United States). Pyramidal neurons were easily identifiable in the slice based on soma morphology and the presence of a prominent apical dendrite. For all electrophysiological recordings, membrane voltages were adjusted for liquid junction potentials (approximately −14 mV) calculated using JPCalc software (P. Barry, University of New South Wales, Sydney, NSW, Australia; modified for Molecular Devices). Signals were filtered at 4–6 kHz through a −3 dB, four-pole low-pass Bessel filter and digitally sampled at 20 kHz using a commercially available data acquisition system (Digidata 1550A with pClamp 10.5 software; Molecular Devices). Data were recorded using pClamp and stored on a computer for offline analysis. Current clamp recordings were analyzed using Clampfit (Molecular Devices). For studies examining synaptic transmission, the amplitude and frequency of miniature excitatory postsynaptic currents (mEPSCs) and miniature inhibitory postsynaptic currents (mIPSCs) were measured using MiniAnalysis 6.0.7 (Synaptosoft; Decatur, GA, United States), and the threshold for mEPSC and mIPSC detection was set at twice the root mean square (RMS) of the background noise.

#### Intrinsic Excitability Measurements

For intrinsic excitability measurements, patch electrodes were filled with a solution containing the following: 130 mM K-gluconate, 10 mM KCl, 10 mM HEPES, 10 mM sodium phosphocreatine, 4 mM MgATP, and 0.3 mM Na_2_-GTP, pH 7.2, 295–300 mOsm. In the current clamp mode, once a stable membrane potential was observed, intrinsic excitability measurements were performed at the resting membrane potential (RMP). Cell capacitance was measured using the membrane test function in pClamp 10.5 (Molecular Devices, Sunnyvale, CA, United States). All measurements of intrinsic membrane excitability were taken from RMP. Rheobase was measured by applying depolarizing current steps (10 pA steps, 100 ms duration) until the generation of a single action potential (AP). Input (membrane) resistance (R_*input*_) was measured by applying a hyperpolarizing current step (−10 pA) via the patch electrode. AP threshold was defined as the *V*_*m*_ measured 0.5 ms before the peak in the second derivative of the waveform. The action potential threshold and amplitude were analyzed for the first spike at the rheobase current injection. AP half-width (AP_50_) was determined by measuring the elapsed time from the peak of the AP to 50% maximum amplitude during the repolarization phase. Firing rate was measured in response to 20 pA depolarizing current steps in the current clamp configuration. The number of action potentials generated over a period of 1 s was recorded across the stimulus intensity range of 0–280 pA. All intrinsic excitability measurements were conducted in oxygenated aCSF at 34–36°C. Cells with RMP lower than −55 mV were included in the final analysis. Recordings were obtained from 14 to 17 cells from three rats in each group.

#### Synaptic Drive Measurements

To measure synaptic drive, miniature postsynaptic currents (mPSCs) were recorded in the presence of TTX (0.5 μM; Hello Bio; Princeton, NJ, United States). Patch electrodes were filled with a solution containing the following (in mM): 130 Cs-gluconate, 10 CsCl, 10 HEPES, 11 EGTA, 1 CaCl_2_, and 2 MgATP, pH 7.2 (295–305 mOsm). In order to isolate mEPSCs, cells were voltage clamped at −70 mV. To record mIPSCs, cells were held at 0 mV. Peak mPSC amplitude was measured from baseline. Decay kinetics were estimated using a single exponential function: [y(t) = a × exp(−t/τ)] using the average mPSC in a given neuron. Synaptic drive was measured in each sampled neuron by multiplying the area under the average mPSC by the mPSC frequency to measure the overall charge transfer across the membrane. mEPSC and mIPSC recordings were obtained from the same 14–18 cells from three rats in each group. For mIPSC recordings, additional recordings were performed from the same three rats to obtain a total of 23–27 cells in each group.

#### Statistical Analysis

All data sets were tested for normality using the Kolmogorov–Smirnoff test. Data were analyzed by unpaired *t*-test when groups were normally distributed. The Mann–Whitney non-parametric test was performed for groups not following a normal distribution. To ensure that a single animal was not driving the differences in measurements, further analysis with nested *t*-test analysis was conducted to confirm our conclusions (Supplementary Table 1). AP firing rate was analyzed by two-way repeated measures ANOVA with SPS and stimulus intensity as factors. In the cases where significant differences and interactions were found, multiple comparisons with false discovery rate correction (FDR) was performed for *post hoc* analysis. Data were analyzed using Prism 8 (GraphPad Software, La Jolla, CA, United States). Outliers for normally distributed dataset were calculated using Prism Grubbs’ test and excluded from the analysis.

## Results

### SPS Reduces the Intrinsic Excitability of Layer V Pyramidal Neurons in the IL mPFC

**FIGURE 1:**
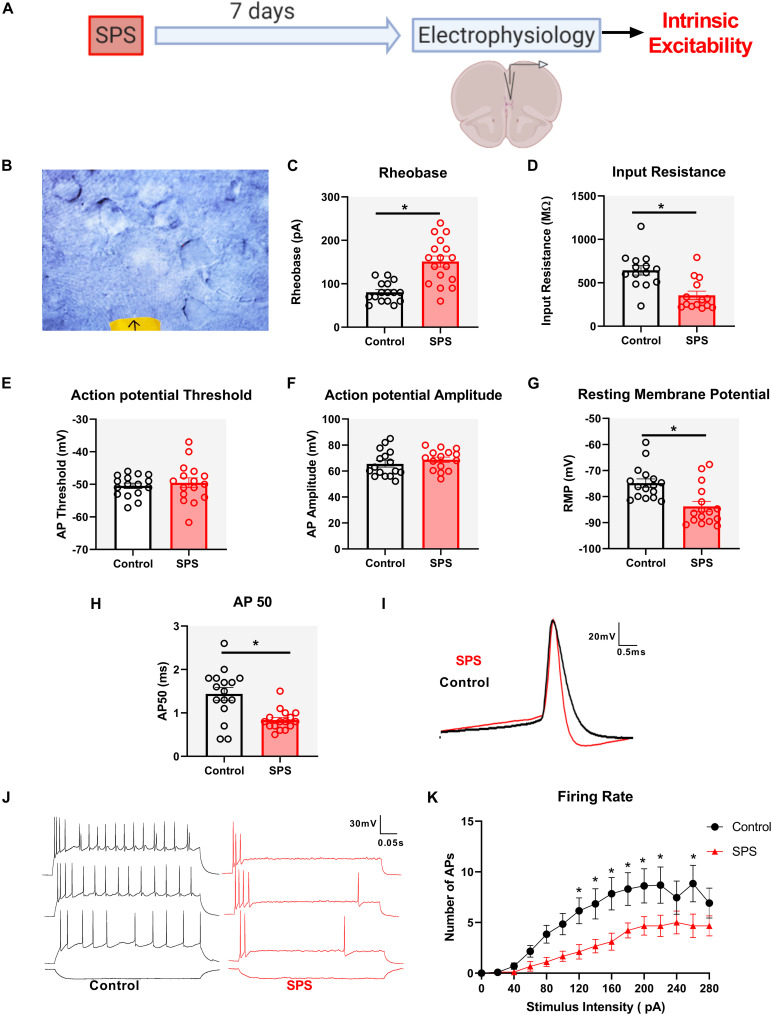
SPS decreases the intrinsic excitability of IL pyramidal neurons. Schematic of the experimental timeline **(A)**. Image of brain slice obtained using warm slicing protocol is represented in **(B)**. Healthy pyramidal neurons are triangular in shape and have thick apical dendrite. Electrophysiological recordings in current clamp mode from the IL mPFC were conducted in male rats 7 days post SPS. SPS increases rheobase **(C)** [*t*(31) = 5.6, *p* < 0.0001], decreases input resistance **(D)** [Mann–Whitney *U*(26) = 29, *p* < 0.01], hyperpolarizes RMP **(G)** [Mann–Whitney *U*(34) = 45, *p* < 0.01], decreases AP_50_ **(H)** [*t*(30) = 3.8, *p* < 0.001] and also decreases firing rate of IL pyramidal neurons **(K)** with a main effect of SPS [*F*(1,20) = 4.7; *p* < 0.05], main effect of stimulus intensity [*F*(14,280) = 20.3; *p* < 0.01] and a significant SPS X stimulus intensity interaction [*F*(14,280) = 2.03; *p* < 0.01]. The SPS group had a significantly lower action potential firing rate compared to controls at a stimulus intensity range of 120–220 pA and at 260 pA (*p* < 0.05). Representative traces of AP50 are shown in **(I)**. Scale bar: 20 mV, 0.5 ms. Representative traces of action potentials in control (black) vs. SPS (red) groups following –40, 100, 160, and 220 pA current injection are shown in **(J)**. Scale bar: 30 mV, 0.05 s. SPS had no effect on action potential threshold **(E)** [*t*(30) = 0.55, *p* > 0.5] or amplitude **(F)** [*t*(29) = 0.96, *p* > 0.5]. Data presented as Mean ± SEM. * indicates *p* < 0.05. For **(B–H)** *n* = 14–17 cells from three rats in each group. For **(J)**, *n* = 13 and 9 cells from three rats in control and SPS group, respectively.

One week following SPS, patch clamp recordings were obtained from layer V pyramidal neurons under the current clamp configuration to probe potential changes in the intrinsic membrane properties of this population (Figure 1A). Experiments were performed on three animals per group. A representative image of the slice is shown in Figure 1B depicting healthy pyramidal neurons with thick apical dendrite. Rheobase was increased in animals exposed to SPS [*t* = 5.6, df = 31, *p* < 0.0001, cell *n* = 16 (control) and 17 (SPS); Figure 1C]. SPS also decreased input (membrane) resistance (*U* = 29, *p* = 0.001, cell *n* = 14/group; Figure 1D), which may contribute to the increase in rheobase observed following SPS. There was a significant decrease in RMP following SPS (*U* = 45, *p* = 0.001, cell *n* = 16/group; Figure 1G). SPS also significantly decreased AP_50_ (*t* = 3.8, df = 30, *p* = 0.0006, cell *n* = 16/group; Figures 1H,I). Mean capacitance between the SPS and control groups were not significantly different (*t* = 0.42, df = 30, *p* = 0.8, cell *n* = 16/group). Mean capacitance of the cells were 90.2 ± 3.4 pF and 94.8 ± 5.6 pF for control and SPS groups, respectively. No significant change in AP threshold (*t* = 0.55, df = 30, *p* = 0.6, cell *n* = 16/group; Figure 1E) or AP amplitude [*t* = 0.96, df = 29, *p* = 0.34, cell *n* = 16 (control) and 15 (SPS); Figure 1F] were observed following SPS.

We next analyzed the repetitive firing rate of the pyramidal neurons following SPS (Figures 1J,K). There was a significant SPS X stimulus intensity interaction [*F*(14,280) = 2.03; *p* = 0.002, cell *n* = 13 (control) and 9 (SPS); Figure 1K]. Multiple comparisons with FDR correction indicate that the SPS group had significantly lower action potential firing compared to the control group at a stimulus intensity range of 120–220 pA and at 260 pA (*p* < 0.05). Collectively, these results suggest that SPS reduces the intrinsic membrane excitability of layer V IL pyramidal neurons.

### SPS Reduces Excitatory Synaptic Drive Onto Layer V Pyramidal Neurons in the IL mPFC

**FIGURE 2:**
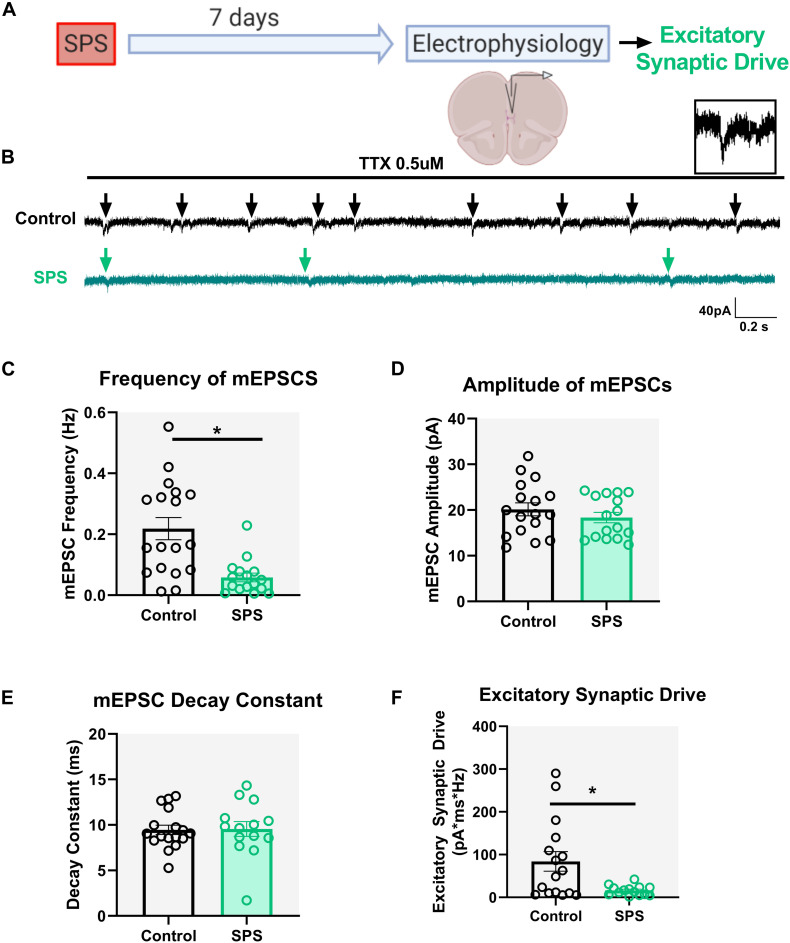
SPS decreases spontaneous glutamatergic drive onto IL pyramidal neurons. Schematic of the experimental timeline **(A)**. Electrophysiological recordings in voltage clamp mode from the IL mPFC were conducted in male rats 7 days post SPS. Representative voltage clamp mEPSC traces of control (black) and SPS (green) groups are shown in **(B)**. Arrows indicate mEPSC events. Scale bars: 40 pA, 0.2 s. Magnified image of a single mEPSC event is shown on top right **(B)**. SPS decreases frequency of mEPSCs **(C)** [*t*(32) = 3.9, *p* < 0.01]. SPS has no effect on mEPSC amplitude **(D)** [*t*(32) = 0.9, *p* = 0.3] or mEPSC decay rate **(E)** [*t*(29) = 0.1, *p* = 0.9]. SPS significantly decreases excitatory synaptic drive **(F)** (Mann–Whitney *U* = 62, *p* < 0.05). Data presented as Mean ± SEM. * indicates *p* < 0.05. *n* = 14–18 cells from three rats in each group.

Previous studies indicate reduced overall glutamate levels in the mPFC following SPS (Piggott et al., 2019), but the underlying mechanisms by which SPS alters glutamatergic transmission in the region remain unclear. Seven days after SPS, mEPSCs were recorded in pyramidal neurons under voltage clamp conditions at holding potential of −70 mV (Figure 2A). Experiments were performed on three animals per group. Our results show that SPS significantly reduces the frequency of mEPSCs [*t* = 3.9, df = 32, *p* = 0.0004, cell *n* = 18 (control) and 16 (SPS); Figures 2B,C] while having no effect on mEPSC amplitude [*t* = 0.9, df = 31, *p* = 0.33, cell *n* = 17 (control) and 16 (SPS); Figure 2D] or mEPSC decay [*t* = 0.1, df = 29, *p* = 0.91, cell *n* = 17 (control) and 14 (SPS); Figure 2E]. Finally, we show that SPS decreases overall excitatory synaptic drive onto pyramidal neurons in the IL mPFC (Mann–Whitney *U* = 62, *p* = 0.03, cell *n* = 16/group; Figure 2F). Collectively these data suggest that SPS reduces spontaneous excitatory synaptic drive onto IL layer V pyramidal neurons, which is likely driven by a presynaptic mechanism.

### SPS Prolongs the Decay of GABA Currents With No Effect on Total Inhibitory Synaptic Drive Onto Layer V IL mPFC Pyramidal Neurons

**FIGURE 3:**
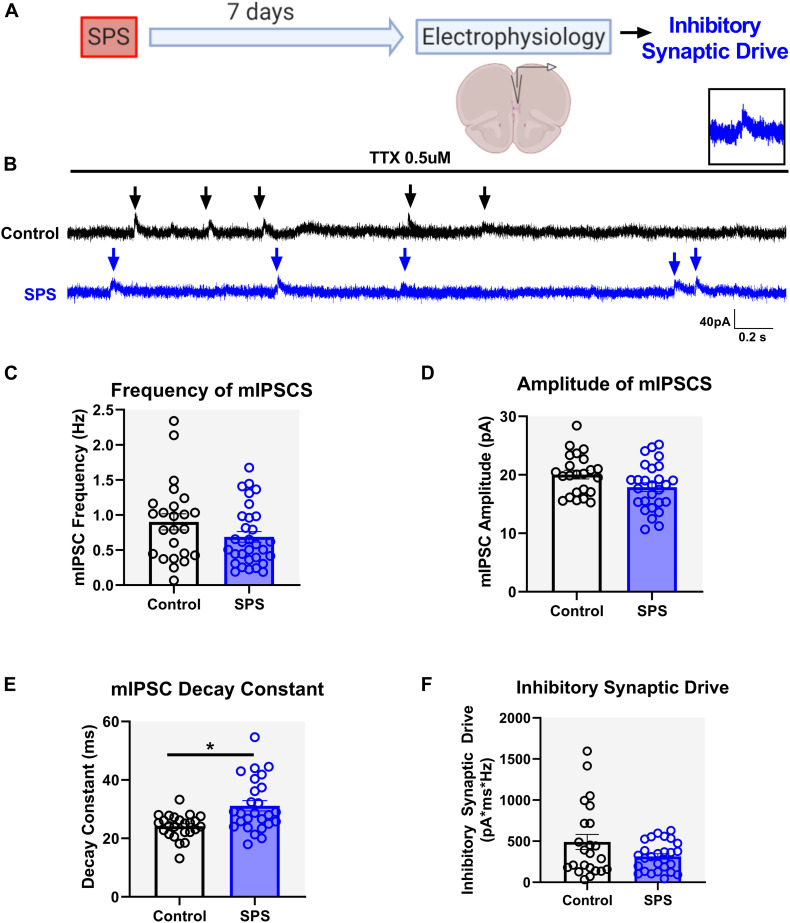
SPS prolongs the decay of GABA currents but has no effect on overall spontaneous inhibitory synaptic drive in the IL. Schematic of the experimental timeline **(A)**. Electrophysiological recordings in the voltage clamp mode were obtained from the IL mPFC in male rats 7 days post SPS. Representative voltage clamp mIPSC traces of control (black) and SPS (blue) groups are shown in **(B)**. Arrows indicate mIPSC events. Scale bars: 40 pA, 0.2 s. Magnified image of a single mIPSC event is shown on top right **(B)**. SPS has no effect on mIPSC frequency **(C)** [*t*(49) = 1.6, *p* = 0.1] or mIPSC amplitude **(D)** [*t*(48) = 2.0, *p* = 0.05]. SPS increases the mIPSC decay **(E)** [*t*(48) = 3.5, *p* < 0.01] but has no effect on inhibitory synaptic drive **(F)** (Mann–Whitney *U* = 263, *p* = 0.3). Data presented as Mean ± SEM. * indicates *p* < 0.05. *n* = 23–27 cells from three rats in each group.

Similar to the excitatory synaptic drive experiments, mIPSCs were measured 7 days following SPS under the voltage clamp configuration at a holding potential of 0 mV (Figure 3A). Experiments were performed on three animals per group. Analysis of mIPSC frequency [*t* = 1.62, df = 49, *p* = 0.11, cell *n* = 24 (control) and 27 (SPS); Figures 3B,C] and amplitude [*t* = 2, df = 48, *p* = 0.05, cell *n* = 23 (control) and 27 (SPS); Figure 3D] did not reveal any significant effects. However, analysis of the mIPSC decay constant showed that SPS significantly prolongs the decay of GABA currents in the IL mPFC [*t* = 3.5, df = 48, *p* = 0.001, cell *n* = 24 (control) and 26 (SPS); Figure 3E]. Nonetheless, analysis of total inhibitory synaptic drive did not reveal any significant difference between the SPS and Control groups [Mann–Whitney *U* = 263, *p* = 0.3, cell *n* = 23 (control) and 27 (SPS); Figure 3F]. Collectively, these data suggest that SPS might not affect the presynaptic release of GABA in the IL, but may allow for GABA to be present in the synaptic cleft longer as demonstrated by the reduction in the decay of GABA currents. However, that effect does not result in significant changes in the strength of spontaneous synaptic inhibition within the IL following SPS.

## Discussion

**FIGURE 4:**
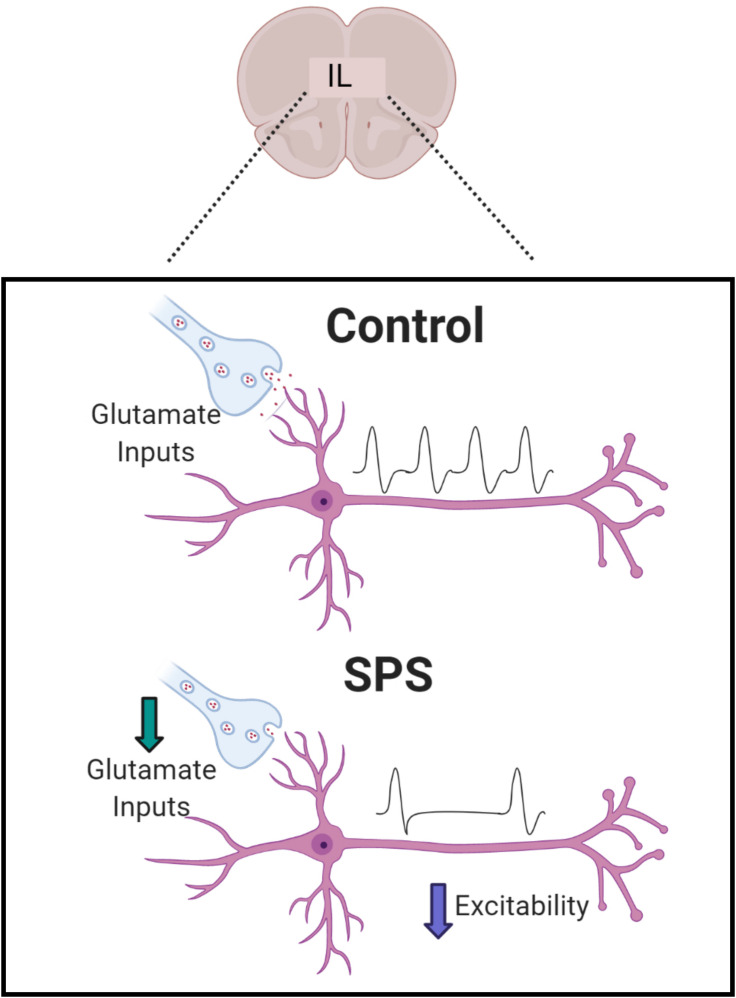
Summary of observed effects. SPS causes a decrease in excitatory synaptic drive and intrinsic excitability of pyramidal neurons which are the major output neurons in Layer V of IL. SPS has been shown to reduce glutamatergic excitatory synaptic drive driven mainly by reduction in presynaptic glutamate inputs. SPS was found to delay the decay of GABA currents but that did not have an effect on overall inhibitory synaptic transmission.

Our results indicate that SPS causes physiological changes in IL mPFC glutamatergic pyramidal neurons and their associated synaptic inputs (Figure 4). Here we demonstrate that SPS reduces the intrinsic membrane excitability of IL pyramidal neurons, as indicated by an increased rheobase, decreased input resistance, hyperpolarized RMP, and a reduction in repetitive firing rate. In addition, SPS causes alterations in spontaneous synaptic drive onto the major output pyramidal neurons in layer V of the IL. SPS reduces the excitatory glutamatergic synaptic tone via presynaptic mechanisms. Our results further indicate that SPS prolongs the decay of GABA currents in the IL but does not change total inhibitory synaptic drive. Collectively, these data suggest possible mechanisms via which SPS may cause a reduction in IL mPFC activity and contribute toward a better understanding of the pathophysiology associated with PTSD symptoms.

Single prolonged stress causes deficits in fear learning and extinction of fear responses (Iwamoto et al., 2007; Knox et al., 2012, 2016; Keller et al., 2015). The IL mPFC in rodents and the ventromedial cortex (Brodmann area 25) in humans plays a role in driving extinction of fear responses (Quirk et al., 2000; Quirk and Mueller, 2008; Milad and Quirk, 2012). Prefrontal hypoactivity and subsequent amygdala hyperactivity leads to a disruption of the top-down executive control of fear responses, and is thought to underlie the abnormal extinction of fear responses in human PTSD patients and also in rodent models of traumatic stress (Quirk et al., 2003; Milad et al., 2009; Piggott et al., 2019). Prior studies indicate that SPS results in reduced activation of the IL mPFC suggesting reduced prefrontal drive, which might underlie the abnormal fear extinction observed in SPS treated animals (Lisieski et al., 2018; Piggott et al., 2019). Our current clamp results indicate that following SPS, the ability to generate an action potential is impaired in IL layer V pyramidal cells (as evidenced by the increased rheobase, reduced input resistance and hyperpolarized RMP). Furthermore, once firing is initiated, SPS animals show a reduction in the number of action potentials across different stimulus intensities compared to controls. Our findings suggest that SPS reduces the intrinsic membrane excitability of glutamatergic pyramidal neurons in the IL mPFC. It is known that fear conditioning can reduce IL pyramidal neuron excitability, and extinction learning reverses the effect (Santini et al., 2008). Future studies are needed to determine if the reduced baseline excitability following SPS exacerbates the decrease in excitability evoked by fear conditioning, leading to fear extinction deficits.

Our voltage clamp experiments show that SPS reduces spontaneous excitatory synaptic drive onto IL pyramidal neurons. Specifically, our findings indicate that SPS reduces the frequency of mEPSCs without causing any change in mEPSC amplitude. According to the quantal theory of neurotransmitter release, a change in quantal amplitude is interpreted as a change in postsynaptic function, whereas a change in quantal frequency is thought to represent a change in presynaptic neurotransmitter release (del Castillo and Katz, 1954; Redman, 1990; Stevens, 1993; Choi and Lovinger, 1997). Therefore, our results suggest that SPS causes a reduction in presynaptic glutamate release onto pyramidal neurons without affecting postsynaptic NMDA/AMPA receptor function. The changes in presynaptic input might be due to changes in the probability of glutamate release or the number of glutamatergic synaptic contacts onto the pyramidal neurons, and further studies are needed to answer that question. Nevertheless, our results are consistent with previous reports showing reduced glutamate levels in the mPFC following SPS (Piggott et al., 2019) and further suggest that the stress-evoked reduction in glutamatergic signaling within the IL cortex may be presynaptically mediated.

It is not known which long-range excitatory inputs, or IL pyramidal neuron outputs, are specifically affected by SPS. Afferent input to the IL includes glutamatergic input from regions implicated in emotional memory, including the BLA, thalamus and ventral hippocampus (vHPC) (Hoover and Vertes, 2007). Importantly, BLA-IL projections are selectively activated during extinction of conditioned fear, and stimulation of the BLA-IL connection facilitates extinction of conditioned fear (Senn et al., 2014). Thus, it is possible that SPS targets glutamatergic BLA-IL connections. Layer V pyramidal neurons in the IL project to various limbic structures such as the BLA and periaqueductal gray (PAG), regions that are known to play a role in modulating fear responses (Cheriyan et al., 2016). Activation of the IL-BLA circuit is needed for proper fear extinction (Cho et al., 2013; Bloodgood et al., 2018), and SPS selectively may reduce activation of IL-BLA circuitry (Piggott et al., 2019). Thus, the reduced excitability of IL Layer V pyramidal neurons may be sufficient to influence downstream circuits in the BLA. Future studies should aim to explore this potential circuit specificity of the physiological effects following SPS.

Processes underlying SPS-induced decreases in IL intrinsic excitability or excitatory synaptic drive remain to be determined. The increase in rheobase, decrease in input resistance and a more hyperpolarized RMP indicates that it is more difficult to depolarize the neuron to spike threshold following SPS. A more hyperpolarized RMP and reduced membrane resistance after SPS could be due to an increase in the number, or conductance, of “leak” K^+^ channels, resulting in a greater K^+^ efflux from the cells and reduced excitability (Honoré, 2007). Rat mPFC pyramidal neurons express KCNQ2 channels (K_*v*_7 voltage gated K^+^ channel), the excessive opening of which might reduce neuronal firing in the PFC following stress (Arnsten et al., 2019). Some forms of KCNQ channels are constitutively active and may contribute toward the leak conductance (Schroeder et al., 2000; Goldstein et al., 2001). Moreover, stimulating KCNQ2 channels in IL mPFC reduces intrinsic excitability of IL pyramidal neurons and reduces fear extinction, while inhibiting these channels enhances fear extinction (Santini and Porter, 2010), further suggesting that disruption of M-type K^+^ currents might underlie SPS induced changes in intrinsic excitability. Several lines of evidence also implicate G-protein gated inwardly rectifying K^+^ (GIRK) channel dysfunction in stress-related alterations in the excitability of prefrontal neurons and psychiatric disorders (Clarke et al., 2011; Victoria et al., 2016). Activation of GIRK channels results in K^+^ efflux, which hyperpolarizes the neuronal RMP and dampens neuronal excitability (Dascal, 1997; Hibino et al., 2010). It is also possible that SPS may increase constitutive GIRK channel activity (Takigawa and Alzheimer, 2002; Chen and Johnston, 2005), leading to membrane hyperpolarization and a reduction in excitability. Our results indicate that SPS decreases AP half-width. Modulation of spike duration can affect neurotransmission by altering Ca_*v*_ channel opening (Rowan et al., 2014, 2016). Since Ca^2+^ entry mainly occurs during spike repolarization (Sabatini and Regehr, 1996, 1997), shorter spike width may lead to less calcium influx in the presynaptic terminal resulting in reduced neurotransmitter release and decreased activation of downstream brain regions. Further studies will be needed to determine the role of specific ion channels in modulating the excitability of IL neurons after chronic stress.

Literature evidence regarding changes in GABAergic signaling following SPS is inconsistent. Some studies using MRS show no change in GABA levels following SPS (Knox et al., 2010; Piggott et al., 2019) in rodents or in humans with PTSD (Rosso et al., 2014; Schür et al., 2016). Our results demonstrate a slower decay of GABA currents following SPS, with no evidence for changes in presynaptic GABA release or postsynaptic GABA receptor expression. Slower IPSC decay could be due to factors such as reduced neurotransmitter uptake and delayed clearance (Overstreet and Westbrook, 2003), slower deactivation time of GABA-A receptors (Schofield and Huguenard, 2007), and changes in GABA-A receptor subunit composition which may alter postsynaptic GABA channel closing kinetics (Haas and Macdonald, 1999).

Taken together, our findings suggest that increasing the intrinsic excitability and glutamatergic synaptic input onto IL pyramidal neurons might be effective in preventing some of the behavioral changes observed with SPS (Figure 4). Indeed, various studies indicate that enhanced top-down control of subcortical regions leads to more efficient control of emotion regulation. fMRI studies in humans have shown that greater prefrontal drive may be a resilience factor in PTSD (Chen F. et al., 2018). Recent studies indicate increased neuronal activation of mPFC in resilient mice following chronic predator or social defeat stress (Adamec et al., 2012). Consistent with this hypothesis, direct optogenetic stimulation of the ventral portion of the mPFC has been shown to promote resilience to social defeat stress (Covington et al., 2010). Overall, our findings indicate that reduced prefrontal drive following SPS may underlie the abnormal fear responses observed with the stress paradigm. Our results highlight novel multifaceted mechanisms by which SPS can cause a reduction in PFC activity, supporting growing evidence that severe stress leads to prefrontal hypoactivity, a characteristic of diseases such as PTSD.

## Data Availability Statement

The original contributions presented in the study are included in the article/Supplementary Material, further inquiries can be directed to the corresponding authors.

## Ethics Statement

The animal study was reviewed and approved by the University of Cincinnati Institutional Animal Care and Use Committee. All animal experiments were carried out in accordance with the National Institutes of Health Guide for the Care and Use of Laboratory Animals (NIH Publications No. 8023, revised 1978).

## Author Contributions

NN, MB, and JH: conceptualization, methodology, software, and writing – reviewing and editing. NN: data collection, analysis, and writing – original draft preparation. MB and JH: supervision. All authors contributed to the article and approved the submitted version.

## Conflict of Interest

The authors declare that the research was conducted in the absence of any commercial or financial relationships that could be construed as a potential conflict of interest.

## Publisher’s Note

All claims expressed in this article are solely those of the authors and do not necessarily represent those of their affiliated organizations, or those of the publisher, the editors and the reviewers. Any product that may be evaluated in this article, or claim that may be made by its manufacturer, is not guaranteed or endorsed by the publisher.
