# Supplementary material for: Single Prolonged Stress Reduces Intrinsic Excitability and Excitatory Synaptic Drive Onto Pyramidal Neurons in the Infralimbic Prefrontal Cortex of Adult Male Rats
Source: Front Cell Neurosci. 2021 Jul 23;15:705660. doi: 10.3389/fncel.2021.705660 (PMC8342808; doi:10.3389/fncel.2021.705660)
Supplement: Supplementary file 1 [file Table_1.DOCX]

|  | **Nested t-test Statistics** |
| --- | --- |
| **Figure 1** |  |
| Rheobase | t=3.6; df=4; p<0.05* |
| Input Resistance | t=3.7; df=4; p<0.05* |
| Action Potential Threshold | t=0.5; df=4; p>0.05 |
| Action Potential Amplitude | t=1.00; df=4; p>0.05 |
| Resting Membrane Potential | t=3.6; df=4; p<0.05* |
| AP 50 | t=3.8; df=4; p<0.05* |
|  |  |
| **Figure 2** |  |
| mEPSC Frequency | t=3.51; df=4; p<0.05* |
| mEPSC Amplitude | t=0.9; df=4; p>0.05 |
| mEPSC Decay Constant | t=0.9; df=4; p>0.05 |
| Total Excitatory Synaptic Drive | t=2.7; df=4;p<0.05* |
|  |  |
| **Figure 3** |  |
| mIPSC Frequency | t=0.5; df=4; p>0.05 |
| mIPSC Amplitude | t=0.5; df=4; p>0.05 |
| mIPSC Decay Constant | t=3.5; df=4; p<0.05* |
| Total Inhibitory Synaptic Drive | t=0.4; df=4; p>0.05 |

Supplementary Table 1: Table demonstrates nested unpaired t-test statistics
